# Woody encroachment and forest degradation in sub-Saharan Africa's woodlands and savannas 1982–2006

**DOI:** 10.1098/rstb.2012.0406

**Published:** 2013-09-05

**Authors:** Edward T. A. Mitchard, Clara M. Flintrop

**Affiliations:** 1 School of GeoSciences, University of Edinburgh, Drummond Street, Edinburgh EH8 9XP, UK; 2 School of Biological Science, University of Edinburgh, Darwin Building, King's Buildings, Mayfield Road, Edinburgh EH9 3JR, UK

**Keywords:** Africa, Advanced Very High Resolution Radiometer (AVHRR), deforestation, Normalized Difference Vegetation Index (NDVI), savanna, woody encroachment

## Abstract

We review the literature and find 16 studies from across Africa's savannas and woodlands where woody encroachment dominates. These small-scale studies are supplemented by an analysis of long-term continent-wide satellite data, specifically the Normalized Difference Vegetation Index (NDVI) time series from the Global Inventory Modeling and Mapping Studies (GIMMS) dataset. Using dry-season data to separate the tree and grass signals, we find 4.0% of non-rainforest woody vegetation in sub-Saharan Africa (excluding West Africa) significantly increased in NDVI from 1982 to 2006, whereas 3.52% decreased. The increases in NDVI were found predominantly to the north of the Congo Basin, with decreases concentrated in the Miombo woodland belt. We hypothesize that areas of increasing dry-season NDVI are undergoing woody encroachment, but the coarse resolution of the study and uncertain relationship between NDVI and woody cover mean that the results should be interpreted with caution; certainly, these results do not contradict studies finding widespread deforestation throughout the continent. However, woody encroachment could be widespread, and warrants further investigation as it has important consequences for the global carbon cycle and land–climate interactions.

## 1. Introduction

We currently have little certainty about the fluxes of carbon in tropical ecosystems: the error bars on estimates of carbon fluxes to and from the land surface are almost as large as the fluxes themselves [1–4]. A number of global and regional studies show that throughout most of the tropics deforestation and degradation are widespread, and the perception is that a net reduction in forest area is occurring across tropical forest, woodland and savanna ecosystems [5–9]. This loss of forests in the tropics is a significant component of anthropogenic CO_2_ emissions [5], though it is currently being more than offset by an observed increase in above-ground biomass in intact forests, likely through a combination of CO_2_ fertilization and regrowth [3,4,10–12].

While it is likely to be true that forest losses exceed forest gains in the tropics, the uncertainties in all the estimation methods used are high [1,9,13] and may be biased towards the detection of deforestation as opposed to woody encroachment or recovery. This bias towards the detection of forest loss is due to three reasons: (i) most monitoring bodies are set up with the purpose of mapping forest losses, so emphasize this in their methods, (ii) the sudden, definite nature of forest loss as opposed to the gradual nature of forest regrowth and (iii) the difficulties of assessing changes in mixed tree–grass systems, where significant increases in canopy cover may not trigger a change in a broad vegetation class. These biases may be exacerbated in Africa, as mixed tree–grass systems dominate (it is the location of two-thirds of the world's savanna [14]); historical ground data are especially rare; and the capacity of environmental and forestry departments to perform mapping tends to be limited, with the majority of remote-sensing-based science being performed by scientists from more developed nations, largely independently of local researchers [15] (though some studies are an exception to this [16,17]).

There is thus no reliable map available showing how woody cover has changed in Africa over the recent past: maps of deforestation, for example [1], explicitly ignore forest gains, and detailed high-resolution analyses are typically available only for small areas [16,17]. The Food and Agriculture Organization of the United Nations produces forest resource assessment (FRA) reports every 5 years, providing country-level statistics; though these are not maps, they may be less prone to the bias towards deforestation as statistics are provided by national governments. The FRA 2010 reports that on average the 49 countries from sub-Saharan Africa lost 0.5% of their forest cover each year from 1990 to 2010 [6]. In the most recent period (2005–2010), seven of the 48 countries reported forest area gains, the others reporting no change or forest loss, with these gains often being due to increases in forest plantations, not the recovery of natural forests. These seven countries are also all small, representing just 0.45% of sub-Saharan Africa's total land area. There are also studies that have analysed land-cover change using high-resolution remote sensing data for small subsets (typically 10 × 10 or 20 × 20 km) located in a systematic grid across the continent, for example the TREES projects [16,18]. From these and other sources of evidence, it is clear that deforestation has dominated, and forest cover has reduced in Africa over the recent past.

However, there is growing evidence that woody encroachment into savannas is occurring widely [19,20]: this study reviews the literature and analyses a satellite time series to suggest that significant forest gains, as well as the well-understood forest losses, are occurring in the continent.

## 2. Evidence of woody encroachment in Africa

We have collated a substantial body of local-scale studies that found increases in tree cover in Africa, as shown by the 16 studies from eight countries listed in table 1. These are widely spread, ranging from west Africa (Ivory Coast [30]) through Central Africa (Gabon [29]; Cameroon [21–23]; Congo [25,26]) to eastern Africa (Ethiopia [27,28]) and South Africa [31–34], and cover a wide range of ecosystems and rainfall levels. In all these cases, either forest is expanding into savanna or savanna woodlands are becoming rapidly woodier.

**Table 1.** Studies finding woody encroachment in sub-Saharan Africa.

country	vegetation type	location	method	results	reference
a. Cameroon	enclosed savanna bordered by young semi-deciduous forests	4°20’ N, 13°43’ E	field inventory of woody species along transects and soil carbon isotope analysis	results suggest a fast, nonlinear advance of forest into savanna; forests <100 years old	[21]
b. Cameroon	forest–savanna mosaic	5°13’ N, 12°30’ E	transects and the analysis of aerial photographs/Landsat	gallery forest encroachment into surrounding savannas at 0.6–2 m yr^−1^ between 1950 and 1990	[22]
c. Cameroon	forest–savanna mosaic; forested in south, savanna with gallery forests to the north	6°0’ N, 12°48’ E	field data used to derive woody-cover to NDVI relationship. Landsat and ASTER for change detection	12.6% of the area showed significant positive change in canopy cover from 1986 to 2000, and 7.8% from 2000 to 2006	[23]
d. Cameroon	as above	6°0’ N, 12°48’ E	change in biomass detected by L-band radar: 1996–2007	significant woody encroachment in Mbam Djerem national park region; deforestation to east of park	[24]
e. Republic of Congo	forest–savanna mosaic	5°02’ S, 11°35’ E	leaf area index measurement, transects perpendicular to ecotone	forest progression into savanna at rate of 1–2 m yr^−1^	[25]
f. Republic of Congo	sharp forest–savanna boundary	4°00’ S, 12°30’ E	transects, soil sampling	carbon isotope analysis suggests forests expanding into savannas at 0.2–0.5 m yr^−1^	[26]
g. Ethiopia	dry savanna in southern Ethiopia	4°28’ N 38°11’ E	vegetation sampling inside and outside enclosures	enhanced grazing causes woody encroachment	[27]
h. Ethiopia	as above	4°50’ N, 39°00’ E	landscape classification	reduction in fire causing rapid woody encroachment and reduction in grass cover	[28]
i. Gabon	forest–savanna mosaic 55 km away from Libreville	0°20’ S, 9°20′ E	^13^C analysis of soil samples to build a chronosequence	forest expansion has occurred at ∼1 m yr^−1^ in coastal Gabon	[29]
j. Ivory Coast	forest islands in savanna woodland	7°25’ N, 5°17′ W	monitoring long-term vegetation plots	rapid reforestation	[30]
k. South Africa	woody savanna, heterogeneous in structure and water availability	Kruger: 24°0’ S, 31°29’ E Eastern Cape: 32°48’ S, 26°50’ E	filed studies combined with aerial photography analysis	kruger: threefold increase in woody cover in mesic savanna, no change in dry savannas. Eastern Cape: tree cover increased from 1% in 1973 to 50% in 2007	[31]
l. South Africa	sub-humid grasslands	28°9’ S′, 29°21′ E	aerial photographs, 1945–2006	tree density increased from 1976 onwards. Tree canopy area increased by 10-fold in 35 years	[32]
m. South Africa	rangelands and abandoned cultivated land	33°16’ S, 27°8’ E	analysis of multi-spectral SPOT images	11.5% increase in ‘slightly eroded (dense bush)’ category 1998–2008	[33]
n. South Africa	savanna woodland, different management types	28°02’ S, 32°12’ E	field transect evaluation and aerial photograph analysis	total tree cover increased from 14% in 1937 to 58% in 2004 in a conservation area, from 3% to 50% in a commercial ranching area, and 6% to 25% in a farmed area	[34]
o. Swaziland	low-veld savanna	26°15’ S, 31°50’ E,	analysis of aerial photographs and ground survey	shrub cover increased from 2% in 1947 to 31% in 1990	[35]
p. Uganda	transition from woody savanna to tall tropical forest	2°04’ N, 31°39’ E	combination of field studies and vegetation index-based satellite change detection	14% increase in woody vegetation over a 14-year period	[36]

It should be noted that we did not attempt to collate the studies finding deforestation or degradation: the aim of this study is to investigate the location of woody encroachment, not to directly assess its magnitude compared with anthropogenic forest loss.

## 3. Coarse-scale analysis of changes in woody vegetation, 1982–2006

The longest-term remote sensing dataset suitable for mapping woody vegetation available annually at a continental scale is the Advanced Very High Resolution Radiometer (AVHRR) dataset, which is available from late 1981 to present. AVHRR sensors have been present on a long series of weather satellites controlled by the National Oceanic and Atmospheric Administration. There are significant difficulties with using this dataset to analyse changes in vegetation, related particularly to changing sensor characteristics, equatorial crossing time, atmospheric conditions and their correction, and calibration. Most of these are believed to have been corrected in the production of a Normalized Difference Vegetation Index (NDVI, a standard vegetation index) product by the Global Land Cover Facility, called the Global Inventory Modelling and Mapping Studies dataset (GIMMS [37–39]). Independent verification of the GIMMS dataset with other higher resolution NDVI datasets (e.g. those from the MODIS and SPOT VEGETATION sensors) available for the more recent past have found good correspondence between the datasets in Africa [38,40,41].

GIMMS gives an estimate of NDVI twice per month from 1982 to 2006; however, NDVI does not relate directly to woody cover, so there are many ways the time series could be analysed. Other studies, for example those looking at changes in the Sahelian grasslands, have typically used the NDVI signal from the wet (growing) season [42,43]. However, this approach gives a proxy of total photosynthetic material over time, which is not what is desired for this analysis: here, we are interested in obtaining a signal from only the woody component of the vegetation in these mixed tree–grass systems. We therefore use the average NDVI of the three-month period with the lowest NDVI, which is typically the end of the driest season. In this period, the grass layer will be dead in the majority of ecosystems, but at least some trees have leaves, either retained from the previous wet season or flushed in preparation for the coming wet season [44,45]. A number of studies have found dry-season NDVI to relate to canopy cover in savanna and woodland ecosystems [23,45,46]. We therefore assume that changes in this minimum NDVI (averaged over three consecutive months in order to reduce artefacts owing to cloud cover or calibration) relate directly to changes in tree cover across 8 km AVHRR pixels in the GIMMS dataset.

We have demonstrated that this technique is successful in detecting woody encroachment based on a site in Mbam Djerem National Park in Cameroon, where we know encroachment of savannas by forest is occurring at a rapid rate [23,24]. This signal can be seen in dry-season NDVI from high-resolution datasets (Landsat and ASTER), and is also replicated in the GIMMS dataset [23]. Crucially, the signal is detectable only when the dry-season NDVI is used, but there is no significant signal in the annual average or wet season NDVI time series [23]. We appreciate this evidence is only from one site, but based on preliminary comparisons of the GIMMS dataset to known areas of encroachment from the references in table 1, it appears to be sensitive to changes elsewhere as well. One exception appears to be West Africa, where owing to different land-use and phenology the signals in the NDVI dataset appeared more related to changes in grass fuel loads than tree cover (P. Mayaux 2013, personal communication), and for this reason the West African region was masked from the analysis.

### (a) Methods

(i) The GIMMS data v. 2.0 (1982–2006) were downloaded for Africa [37]. These are pre-processed and corrected NDVI datasets, and were used in the native Albers equal area projection. All analysis was performed using IDL-ENVI v. 4.8 (Exelis).
(ii) Mean NDVI was calculated for every possible consecutive three-month period for each pixel from 1982 to 2006. Only three-month periods where five out of six possible observations reached this ‘best-quality’ standard were considered. Then, the minimum three-monthly NDVI was extracted for each pixel for each year from 1982 to 2006.
(iii) Linear regression was performed across each time series for each pixel. Change in woody vegetation considered to have occurred for all areas with a ‘significant’ best-fit line (using an *F*-test with a 90% confidence level [42] and a slope larger than 0.002, which suggests a change of 0.05 NDVI units in total over the 25 year period). These thresholds are arbitrary and were chosen based on the literature and visual assessments of the maps—they could be refined given a better ground dataset, but are thought to represent areas where there is a strong signal in the data.
(iv) In order to remove areas where the signal came from grasses, pixels containing no ‘wooded’ classes in the Global Land Cover 2000 (GLC 2000 [14]) dataset [17] were removed from the analysis; similarly, this methodology produces spurious results over intact rainforest, with results related to cloud-cover contamination and phenology, so pixels of the ‘closed evergreen lowland forest’ in GLC 2000 were also masked.

### (b) Results and uncertainties from GIMMS analysis

**Figure 1.**
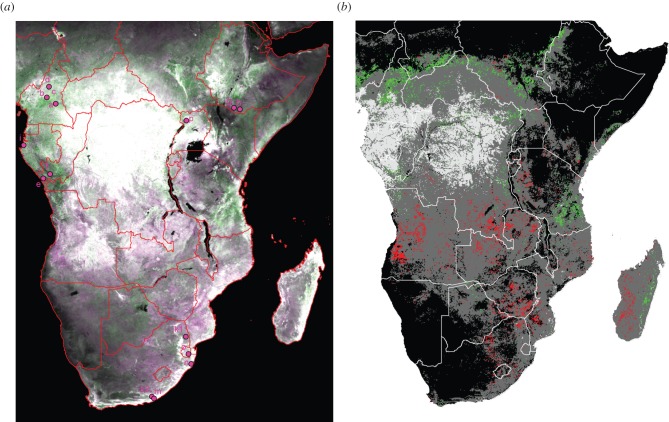
(*a*) The location of studies finding woody encroachment listed in table 1 (a–p), overlaid on the GIMMS dataset with average three-monthly minimum NDVI from 1982 to 1986 in magenta and from 2002 to 2006 in green. (*b*) Areas of significant increasing NDVI trends are shown in green, significant decreasing trends in red. Pixels with no woody vegetation according to Mayaux *et al.* [17] are dark grey, pixels that are ‘lowland evergreen broadleaved forest’ are light grey.

The analysis shows that woody encroachment and forest loss are both occurring (figure 1). Of non-rainforest woody areas, 4.00% showed a significant positive change in NDVI, and 3.52% showed a negative trend (table 2). There is a north–south divide clearly visible: the majority of the increase in woody vegetation is occurring to the north of the equator, with the majority of forest loss detected occurring to the south, especially in Miombo woodland regions.

**Table 2.** Area of sub-Saharan Africa (excluding West Africa) showing significant changes in NDVI.

class	area (km^2^)	% area
**no woody vegetation present (2000)**	**6 819 968**	
**woody vegetation present (2000)**	**10 086 656**	
closed evergreen forest (2000)	1 345 088	
no significant change (1982–2006)	8 084 160	92.48
significant positive NDVI slope (1982–2006)	349 440	4.00
significant negative NDVI slope (1982–2006)	307 968	3.52

These results should be interpreted with caution for a number of reasons: (i) the resolution is very coarse (8 km), meaning that many small-scale deforestation and regrowth events will have been missed: only changes occurring over a significant portion of the pixel will be detected (though it should be noted that despite most 8 km pixels being ‘mixed pixels’, i.e. containing a number of vegetation classes, the results should be robust if the ratio of forested to non-forested vegetation in the pixel changes significantly). (ii) The time series of NDVI data may contain artefacts, particularly over tropical regions, owing to the resampling and cloud-filtering algorithms applied to the raw AVHRR data [47]; though this should be mitigated by the extensive processing undergone by the GIMMS dataset [38,39], no independent verification is available for the critical earlier half of the time series. (iii) The assumption that dry-season mean NDVI relates to woody cover has not been fully validated across the continent, and is likely to lead to errors in some locations, as tree and grass phenology patterns do change across the many ecosystems in this analysis. (iv) Rainfall patterns have changed, and some of the pattern seen could be owing to wetter or drier conditions, leading to different amounts of green vegetation being left in the driest season; this is quite likely in the Sahel region where rainfall has increased significantly over the study period [42].

It is hard to discount the above concerns, but some confirmation comes from the literature review that gave specific instances of the location of woody encroachment. Figure 1*a* locates the studies listed in table 1 on a map, displayed on the NDVI data from the first and final 5-year sections of the time series. All 16 studies were found on pixels that showed a positive NDVI trend over the series, and seven of these 16 were found on pixels where this trend was identified as significant using the criteria in §3*a* (iii).

## 4. Discussion

From both the literature review and the GIMMS analysis, it is clear that both forest loss and gain are occurring widely throughout Africa. In terms of area, it appears that the area of land undergoing woody encroachment may be comparable or even larger than areas where a significant loss of forest cover is occurring; subsetting the GIMMS analysis most of the increase is in the woodland and savannas of sub-Saharan Africa north of the equator, whereas in the Miombo woodland regions, south of the equator, forest loss appears to be dominating. However, this conclusion has high uncertainties owing to potential artefacts in the GIMMS dataset, and regional variation in the relationship between NDVI and woody cover. The results presented here are not directly comparable with analyses based on detailed interpretation of small subsets [16,18], as those interpretations assess changes in vegetation classes, whereas the GIMMS approach sees changes in woody cover aggregated across all vegetation types at an 8 km pixel size. While in places the GIMMS approach and Bodart *et al.* [16] agree, for example finding increases outweighing decreases in the far west of the Democratic Republic of Congo (DRC), the Central African Republic and Ethiopia, and rapid forest lost in the Miombo woodlands of southern DRC, Angola, Zambia, Zimbabwe and Mozambique, in many areas, the GIMMS approach sees more forest gains than Bodart *et al.* [16].

It should be noted that this analysis relates mainly to changes in broad canopy cover in mixed tree–grass areas and does not relate directly to the carbon balance of the African continent (though forest regrowth must form part of the land–surface carbon sink [4]). In particular, this analysis will not see changes in tropical forests, and even in mixed tree–grass systems canopy cover does not relate directly to carbon stocks.

This analysis validates the observations made that Miombo woodlands are suffering especially badly from the loss of woody vegetation, owing to expanding populations removing trees for agriculture and fuel (including charcoal) [48]. That this loss was not shown to have occurred to the same extent in Malawi and Kenya, two areas where the savannas are known to have had their tree density greatly reduced over the past century, may be because much of the damage was already done before the start of the analysis in 1982 [6,49]. The forest loss in Miombo represents a sharp contrast to the gains observed in northern and Central Africa; but, in turn, at least some of this increase may represent a recovery following previous forest loss.

### (a) Causality of forest expansion

To understand the causes of forest expansion, it is necessary to comprehend the current and historical constraints on woody cover throughout the region. It is known that much of the African continent exists currently at a woody cover level far below its potential given its annual rainfall [50,51]. Rainfall is believed to control the maximum possible woody cover in a site up to about 650 mm, but above that point full canopy closure is possible [51]. A large number of factors operate to maintain forest cover at its supressed state, thought to principally be fire (anthropogenic and natural) and grazing. Woody encroachment can therefore be caused by increases in rainfall in drier savanna ecosystems, but in most cases will be caused by changes in the factors that suppress woody vegetation. In particular, it is thought that anthropogenic changes in the fire and grazing regimes may have had significant impacts, potentially supplemented by changes in the climate, in particular the atmospheric CO_2_ concentration.

It is hard to underestimate the anthropogenic influence on Africa's forest cover. Humans have been setting fires and controlling grazer numbers throughout their evolution, potentially even having a major part in the spread of savanna vegetation [52]. In general, it is thought that anthropogenic actions tended to reduce forest cover [50,51], though there is some evidence to the contrary [53]. Changes in the fire regime can have dramatic and rapid effects on increasing or decreasing woody cover [54]. There are also complex interactions at play: for example, the recent expansion of cattle ranching leading to increased grazing pressure can, in fact, cause woody encroachment, by reducing grass fuel load, resulting in a decline in fire frequency and severity, thus reducing sapling mortality and enhancing woody encroachment [35]. Encroachment can even be enhanced by the expansion of road networks (typically thought of as a cause of deforestation), by creating firebreaks [55].

However, several studies suggest that global factors, in particular atmospheric CO_2_ enrichment, are equally important [3]. An increase in atmospheric CO_2_ reduces the advantage held by C_4_ grasses over C_3_ trees: C_4_ grasses use a specialist mechanism to increase the CO_2_ concentration in cells that perform the light reaction of photosynthesis, reducing the rate of photorespiration that is a major limitation on photosynthetic efficiency in high temperatures [56,57]; as the atmospheric CO_2_ concentration increases, this specialist adaptation is less of an advantage. In particular, increased CO_2_ concentrations mean that trees can grow faster and saplings are more likely to be able to grow enough between fires to escape the flame zone [58].

## 5. Conclusions

This study brought together a body of evidence suggesting woody encroachment is widespread in sub-Saharan Africa. The reason behind this encroachment is likely to be a combination of changes in the fire regime and increasing atmospheric CO_2_ concentrations, but further studies will be needed to determine this with more confidence. A coarse-scale analysis of changes in woody vegetation from 1982 to 2006 suggested that significant woody encroachment is occurring to the north of the Congo Basin, but, in contrast, to the south of the Congo Basin a rapid reduction in woody vegetation is occurring. This deforestation in the Miombo woodlands of Africa warrants much more global attention, as it represents a serious threat to the livelihood of the region's many inhabitants and to this unique ecosystem.

The results of this study should be interpreted with caution: the evidence brought together is a collection of small-scale studies, and a coarse-scale remote sensing analysis that can detect only broad changes in woody cover, and is prone to artefacts. These results should stimulate discussion on woody encroachment, but this analysis does not provide a definitive assessment of the total magnitude of woody encroachment compared to forest loss.

## Data accessibility

GIMMS data were provided free of charge by the Global Land Cover Facility at the University of Maryland.
